# Impacts of vessel noise on Red Drum (*Sciaenops ocellatus*) spawning choruses in Saint Andrew Bay, Florida, U.S.A.

**DOI:** 10.1371/journal.pone.0344649

**Published:** 2026-09-09

**Authors:** Bennett H. Price, Dakota Brunetti, Amanda Kirkland, T. Erin Cox, Kelly S. Boyle

**Affiliations:** Department of Biological Sciences, University of New Orleans, New Orleans, Louisiana, United States of America; MARE – Marine and Environmental Sciences Centre, PORTUGAL

## Abstract

Noise pollution is an increasing threat to soniferous fishes, however, research on noise pollution impacts is limited to few species and rarely studied *in situ*. Red Drum (*Sciaenops ocellatus*) is an estuarine, fishery species that choruses during spawning. We tested predictions of the hypothesis that Red Drum alter sound production in response to vessel noise. We used passive acoustic monitoring in 2021 and 2022 at an estuarine inlet and Generalized Least Squares (GLS) models to assess vessel equivalent continuous sound levels (L_eq_) and other abiotic parameters on Red Drum chorus L_eq_. GLS models of daily crepuscular choruses indicated a significant reduction of 0.05dB in Red Drum L_eq_ in relation to every 1dB of crepuscular vessel noise in 2021 (p = 0.026). GLS models testing influence of abiotic variables and prior vessel noise, predicted reduced chorus L_eq_ proportional to prior noise L_eq_: ca. 0.05dB and 0.04dB reduction for every 1dB of vessel L_eq_ in 2021 and 2022, respectively. In some instances, L_eq_ during vessel noise was lower than fish chorus L_eq_ immediately prior, indicating instances when fish reduced chorus amplitude during vessel noise or fled the immediate area. In cases when L_eq_ of vessel noise periods exceeded fish calling L_eq_ immediately prior, it is not known if fish modulated calling amplitude because the portion of combined vessel noise and fish chorus amplitude from vessels is unknown. In peak spawning season (September-October) vessel noise was frequent, detected in >31% of recordings in both years and up to 100% of recordings on some dates. Observations of disrupted choruses and high vessel noise prevalence suggest spawning behavior may be impacted by abundant vessel noise.

## Introduction

In aquatic environments, noise pollution affects animal behavior, hearing, and physiology [1–5]. Shifts in behavior include changes in interactions among conspecifics, fight-or-flight response, and modification of acoustic signaling [6–9]. Over recent decades, anthropogenic noise has steadily increased in coastal marine habitats, such as bays and estuaries, with commercial and recreational boat traffic noise representing a major component of this pollution and small watercraft being a ubiquitous source of noise pollution in coastal waters [3,10,11]. Additionally, sound travels farther in water than in air and thus the region of impact of noise pollution is quite large, particularly from sources of low frequency sound [12]. Vessel noise is often low frequency and overlaps with the call spectra and bandwidth of hearing of many fish species [3,13,14], and it is currently believed that all extant fish species can detect low frequency sound [15–17].

Sound production in fishes occurs in courtship, disturbance, territorial displays and other unknown contexts [18–20]. Disruption of courtship and spawning holds obvious implications for organismal fitness and thus it is important to determine if noise pollution negatively influences these behaviors. Sciaenidae (croakers and drums) present an excellent family for research on impacts of vessel noise on sound production behavior. Sciaenid sound production is associated with spawning and disturbance [19]. Furthermore, members of this family play key ecological roles in estuarine food webs and support economically significant fisheries in the U.S. and globally [21–24]. Red Drum (*Sciaenops ocellatus*) is a soniferous sciaenid species in which calling is strongly correlated with reproduction [20,25,26]. In the Gulf of Mexico, Red Drum are protected in federal waters and suspected to be overfished, but stock data for Red Drum are limited within this region [27]. Call spectra and hearing range of Red Drum are both low frequency, with dominant frequency of calls between 100–200 Hz and hearing most sensitive from 100–500 Hz [14,28–30].

During summer and autumn, Red Drum form large, high-density spawning aggregations associated with intense choruses made by male fish in the afternoon into the evening and peak from August through October [20,26]. Notably, vessel noise can occur in the same habitat and overlap temporally and in the sound spectrum with Red Drum calls [31]. This overlap in vessel noise and call spectra presents a potential risk for acoustic communication with implications for spawning success if fish reduce or terminate calling during vessel noise or if fish continue to call when their signals are unable to be received by conspecifics.

**Fig 1:**
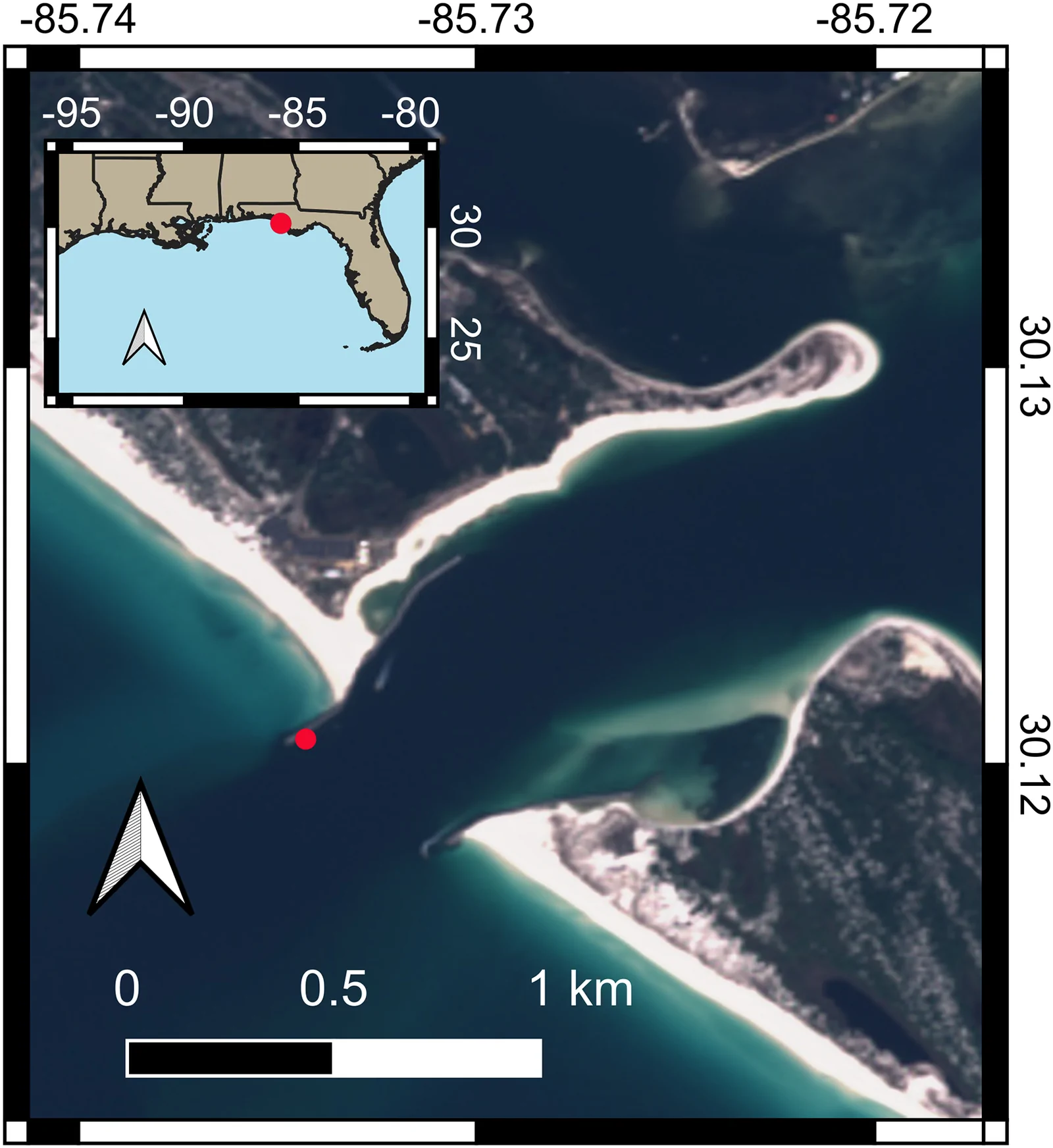
Saint Andrew Bay, Florida, U.S.A. The red star notes placement of the hydrophone on the southwestern end of the jetty at the mouth of the bay (Contains modified Copernicus Sentinel data 2026 processed by the Copernicus Data Space Ecosystem.).

Our study tests for influence of noise pollution on Red Drum sound production behavior in a busy navigation channel in the northern Gulf of Mexico at Saint Andrew Bay in Panama City Beach, Florida, U.S.A. (Fig 1). This field site was chosen because both mature Red Drum and frequent vessel traffic were observed in this bay by the authors. Saint Andrew Bay is located in the greater Panhandle region of western Florida, where both Red Drum stocks and fishing pressure have been increasing, with fishing effort doubling in this area over the past decade and 2023 having the highest abundance of young of year Red Drum in over 20 years [32].

We hypothesize Red Drum alter their calling when vessel noise co-occurs in St. Andrew Bay. We considered two alternative outcomes for how soniferous fish may respond in the presence of vessel noise. First, the disturbance hypothesis predicts that fish calling behavior is disturbed by vessel noise. Predictions of this hypothesis are that fish reduce or completely cease calling during and shortly after vessel noise exposure. Consistent with this prediction, in previous studies, a reduction in calling behavior was observed in Sciaenidae and Gobiidae in association with vessel noise [8,9,33]. Additionally, overall spawning success was lower in gobies exposed to noise pollution [8], and in Red Drum, spawning was only observed with increased drumming behavior [20,25]. Fish can also flee the noisy area which would result in a reduction of observable drumming in the immediate habitat and be consistent with the disturbance hypothesis. As such, reduction in sound production due to a disturbance response may adversely impact reproductive success. Alternatively, we considered a compensation hypothesis that predicts fish will increase or alter calls to compete with higher levels of background noise. This behavioral shift of calling level or dominant frequency in the presence of excessive background noise is known as the Lombard Effect [34,35]. Beyond these two predicted outcomes there may also be no-modification of fish sound production observed in the presence of vessel noise. No-modification of behaviour during intense noise could represent wasted signaling effort could occur if fish do not modify sound production effort or even if the Lombard Effect occurs but is not sufficient to compete with background noise.

We used passive acoustic monitoring to measure the response of Red Drum to vessel noise to test predictions of compensation, disturbance, and no-modification in calling. We examined Red Drum calls and choruses and vessel noise during a two-hour crepuscular period over two spawning seasons (July-October, 2021 and 2022). We investigated whether: (1) cumulative exposure of prior vessel noise L_eq_ impacts fish sound production, (2) if fish alter sound production following individual vessel events, and (3) whether fish alter sound production during vessel noise exposure that co-occurs with fish calling. Additionally, (4) we tested if abiotic variables (e.g., water temperature, lunar phase) were predictive of Red Drum calling in conjunction with vessel nose and how this might affect vocal behavior over the spawning season and (5) documented the prevalence of vessel noise co-occurring in the same habitat as Red Drum calling.

## Materials and methods

### Ethics statement

No Institutional Animal Care and Use Committee approval was required for this study as no manipulation, sampling, or collection of any animals was done as part of experimental protocols. Approval of field site use was granted by the Florida Department of Environmental Protection (permit # 02252022011), Saint Andrews State Park.

### Data collection and field site

The inlet of Saint Andrew Bay, Panama City Beach Florida (Fig 1), was monitored via a single passive acoustic monitor (SNAP recorder, 2dB gain, 44.1kHz, Loggerhead Instruments, Sarasota, FL, U.S.A., http://www.loggerhead.com) with hydrophone sensitivities of recorders that ranged from −169.8 to −170.5 dB re 1V/µPa. Recordings took place during the Red Drum spawning seasons (July-October 2021 and 2022). A chain secured the recorder to a large boulder at a depth of ~9 m. This site faced the navigation channel in the bay and was observed to be heavily trafficked by both commercial and recreational watercraft with maritime traffic density averaging 26.6 monthly hours per km^2^ during our recording months in 2021 and 24.9 monthly hours per km^2^ in 2022 [36]. The recorder was set to a 15% duty cycle recording a 45 second file once every 5 minutes. A dive team swapped out the recorder approximately every 45 days throughout the spawning seasons, replacing it with a fresh recorder. A continuous recording, in place of a duty cycle recording, was made during October 2022 via a SNAP recorder installed in the same location (St. Andrew Bay Pass). The recorder was left to record 45 second files with no pauses until the end of battery life (5.25 days).

#### Abiotic data.

Abiotic data were used to assess what environmental factors beyond noise pollution predict Red Drum vocal behavior. For each SNAP deployment an Onset HOBO data logger was deployed at the same location. Hourly temperature readings were averaged by calendar day (0:00–23:59) for a daily value in analysis. Other predictive variables used were daylength, tidal events (time of high tide and low tide, tide differential) for each 24h period (0-24h), time of moonrise, moon phase (illumination %), and time the moon crossed the meridian [37]. If no tidal event occurred in a 24 h period (0-24h calendar day), the next proceeding event was used (24h + difference between events). If two tidal events occurred in the same 24 h period, the highest differential was used.

#### Screening recordings.

A two-hour crepuscular period (CP) (30 min prior to and 90 min post sunset) was visually and aurally screened with Raven Lite 2.05 software [38] and categorized based on presence/absence of vessel noise and Red Drum calls for each day through both spawning seasons. The CP was chosen for analysis because it encompasses the period when Red Drum spawning activity is most prevalent [20,26]. The CP was analyzed for both duty cycle and continuous recordings with all files bandpass filtered in R [39] using the Seewave package [40]. A 7^th^ order Butterworth filter was used to isolate the 0–0.6 kHz band. This frequency band was chosen because these frequencies encompass the dominant bandwidth of Red Drum calls and hearing capabilities [28,29]. We then calculated root mean square (RMS) band (0−0.6kHz) sound pressure level (SPL dB re: 1 µPa) for each 15 s interval of the file, which was then averaged for each 45 s sound file. Recordings with noise artifacts from intense weather conditions, such as hurricanes, or the hydrophone being bumped or jostled were not used for analysis. Daily background noise levels were calculated to account for biotic and abiotic variation of background noise. This was done using files from within the CP that contained neither detectable vessel nor Red Drum sounds, though other biotic sounds may still be present in these recordings as they do contribute to natural variation in background noise levels. If full recordings without vessel or Red Drum sounds were not available in the CP, a minimum period of five seconds without vessel noise or Red Drum calling was sampled within a file. If no such period existed within the CP a 5s period was sampled either immediately prior or after the CP to estimate evening background noise levels.

Received SPL was used as a measure of fish calling as counting calls was untenable when fish chorused or when vessel noise obscured the soundscape. SPL of each sound source (Red Drum, SPL_fish_; vessel noise, SPL_vessel_) within a file was determined in the following manner. Estimated SPL contribution of the sound source to the 0−0.6kHz band sound pressure level of the file was calculated using the power summation equation, equation 11 of Ma et al., (2005) [41] and subtracting the estimated contribution of evening background noise from the total band RMS sound pressure level of the file. Vessel noise SPLs may also contain Red Drum sound, but it was not possible to directly count calls or estimate the contribution of Red Drum calls when vessel noise obscures potential fish calls. We explored this possibility below (*see Immediate impacts of vessel noise during fish calling)*. In some cases, SPLs in the 0−0.6kHz band were lower than estimated background noise levels (likely due to limits in precision of background noise estimates). These cases were relatively rare (10% of files with vessel noise in 2021, 9% of files with vessel noise in 2022, 16% of files with fish calls in 2021, and 13% of files with fish calls in 2022) and in these instances, SPLs of vessels or fish were estimated as zero. We estimated total contribution of fish calls and vessel noise of each CP as a sound level equivalent (L_eq_) (dB re: 1μPa). L_eq_ of fish calls for each CP was estimated using methods from Rice et al., (2014) [42] as follows:


$$\begin{array}{c} {\text{Leq}\;}_{\text{fish}}\text{ dB re}:\;\text{1}\;\mu {\text{Pa}}^{\text{2}}\cdot \text{s}=\text{1}\text{0}{\text{log}}_{\text{1}\text{0}}\left(\sum_{k=\text{1}}^{n}{\text{FS}\text{E}}_{k}\right)-\text{1}\text{0}{\text{log}}_{\text{1}\text{0}}\left({\text{Env}}_{\text{cp}}\right) \end{array}$$ (1)

The number of files with fish calling is represented by *n. FSE* is the sound pressure level of a single 45 s file, estimated by squared SPL_fish_ (µPa^2^). *Env*_*cp*_ is the number of files without vessel noise present for the whole CP (*Env*_*cp*_ ≤ 24). Thus, *Env*_*cp*_ accounts for the amount of time (CP or portion of CP without vessel noise) for which L_eq fish_ is estimated. Thus, L_eq fish_ accounts for sustained levels of FSE across the CP. For example, a single instance of fish calling at 129 dB re: 1µPa^2^ out of 24 files would be approximately equal to 24 instances of a fish calling at 101 dB re: 1µPa^2^, ~ 115 dB re: 1µPa^2^ ⋅ s.

Vessel noise L_eq_ was calculated in a similar manner:


$$\begin{array}{c} {\text{Leq }}_{\text{vessel}}\;\text{dB}\;\text{re}:\;\text{1}\;\mu \text{Pa2}\cdot \text{s}={\text{10log}}_{\text{10}}\left(\sum_{k=\text{1}}^{m}{\text{VSE}}_{k}\right)-{\text{10log}}_{\text{10}}\left({\text{E}}_{\text{cp}}\right), \end{array}$$ (2)

where VSE is the estimated SPL in µPa^2^ of a sound file with vessel noise present, *m* is the total number of files with vessel noise for the whole CP on that date, and *E*_*cp*_ is the total number of files of the CP on that date.

Files from continuous recordings were visually and aurally screened and categorized as above. When vessel noise was detected, recordings were band pass filtered (0–600 Hz) as described above and the band pressure of filtered files was estimated from RMS amplitudes measured in Audacity software to determine SPL within specific periods of time relative to vessel noise occurrence (see section Fish calling during and immediately following individual vessel noise events, below).

#### Spatial and temporal overlap of vessel noise and red drum choruses.

For each CP we tallied the total number of recordings with either vessel noise or Red Drum calls without any vessel noise present. From these values, for each daily CP we calculated ‘fish calling tendency’ (fct), ‘vessel noise portion’ (vnp), ‘noise-free fish calling portion’ (n-ffcp), and the noise free portion (n-fp), as the following:


$$\begin{array}{c} fct=nFish/\;nVNfree*\text{1}\text{0}\text{0}, \end{array}$$ (3)

$$\begin{array}{c} vnp=nVN/nCP*\text{1}\text{0}\text{0}, \end{array}$$ (4)

$$\begin{array}{c} n-ffcp=nFish/nCP*\text{1}\text{0}\text{0}, \end{array}$$ (5)

$$\begin{array}{c} n-fp=nVNfree/nCP*\text{1}\text{0}\text{0}, \end{array}$$ (6)

Where,

*nFish* - number of files with fish calls but no vessel noise,

*nVNfree* – number of files without vessel noise,

*nVN* - number of files with vessel noise,

*nCP* - number of files in the CP,

We expected the fct to reflect calling propensity changes that parallel seasonal changes in spawning activity. Values of vnp likely follow seasonal patterns of vessel traffic in the area and the n-ffcp reflects the amount of the CP occupied by fish calls without vessel noise as a combination of fct and the size of the n-fp, in other words, the percentage of the crepuscular period in which fish called while vessel noise was not present. The nfp reflects the percentage of the crepuscular period that lacked vessel noise. For both seasons, we summarized the monthly mean, median, quartiles, interquartile range, and outliers of the fct, vnp, n-ffcp, and n-fp.

#### Effects of vessel noise and other abiotic variables on fish calling.

We tested the combined influences of vessel noise and potential environmental predictor variables on fish calling L_eq_ over the CP among dates. To determine which predictor variables had the greatest influence on fish calling L_eq_ by evening, complex Generalized Least Square (GLS) models were constructed in R package nlme [43]. GLS models are ideal for time series data where autocorrelation between residuals is likely to occur. Initial models included all abiotic measurements (vessel noise L_eq_, water temperature, daylength, time of high tide, time of low tide, tide differential, moon phase, moonrise time, and time of moon passing the meridian). Date was also included as a predictor and coded with consecutive integers beginning with 1 for 1 July. Additionally, an interaction of date and daylength was included as a predictor, as daylength shortened throughout the months inversely with increasing date. Red Drum spawning season ends prior to the shortest day of the year. These models were simplified in a backwards stepwise fashion to obtain the combination of variables which produced the lowest Aikake Information Criterion value corrected for small sample size (AICc). From the best fit model, predictor variables were removed if their inclusion raised the model <2  AICc units [44]. For these models we also tested for serial autocorrelation [45] based on the expectation that fish calls may cluster among consecutive days. We conducted backwards stepwise model selection (as described above) for the first (AR1) and second order (AR2) autoregressive models and a non-autocorrelation (non-AR) model. We then selected the best model among these three models and three null models (non-AR, AR1, and AR2) based on AFICc.

#### Prior exposure impact on Red Drum calling.

To test whether prior vessel noise exposure affects fish calling later in the evening, we used GLS models to determine whether fish calling L_eq_ in the final 15 min. portion of the CP is predicted by vessel noise exposure in the prior 105 min. of the CP. Although Red Drum calling may be less intense in the final portion of the CP, we chose to examine this period in order to determine if prior vessel noise influences fish calling. Thus, our analysis compared fish calling among days at the same time point relative to sunset. In addition to prior vessel noise, the same abiotic variables used to test fish calling L_eq_ over the CP were used in initial GLS models that were simplified step-wise (as described above). CPs with no fish calling present in the final 15 min. of the CP were not included in this analysis as it was unknown if fish were still present in the recording area.

#### Fish calling during vessel noise compared to periods without vessel noise.

**Fig 2:**
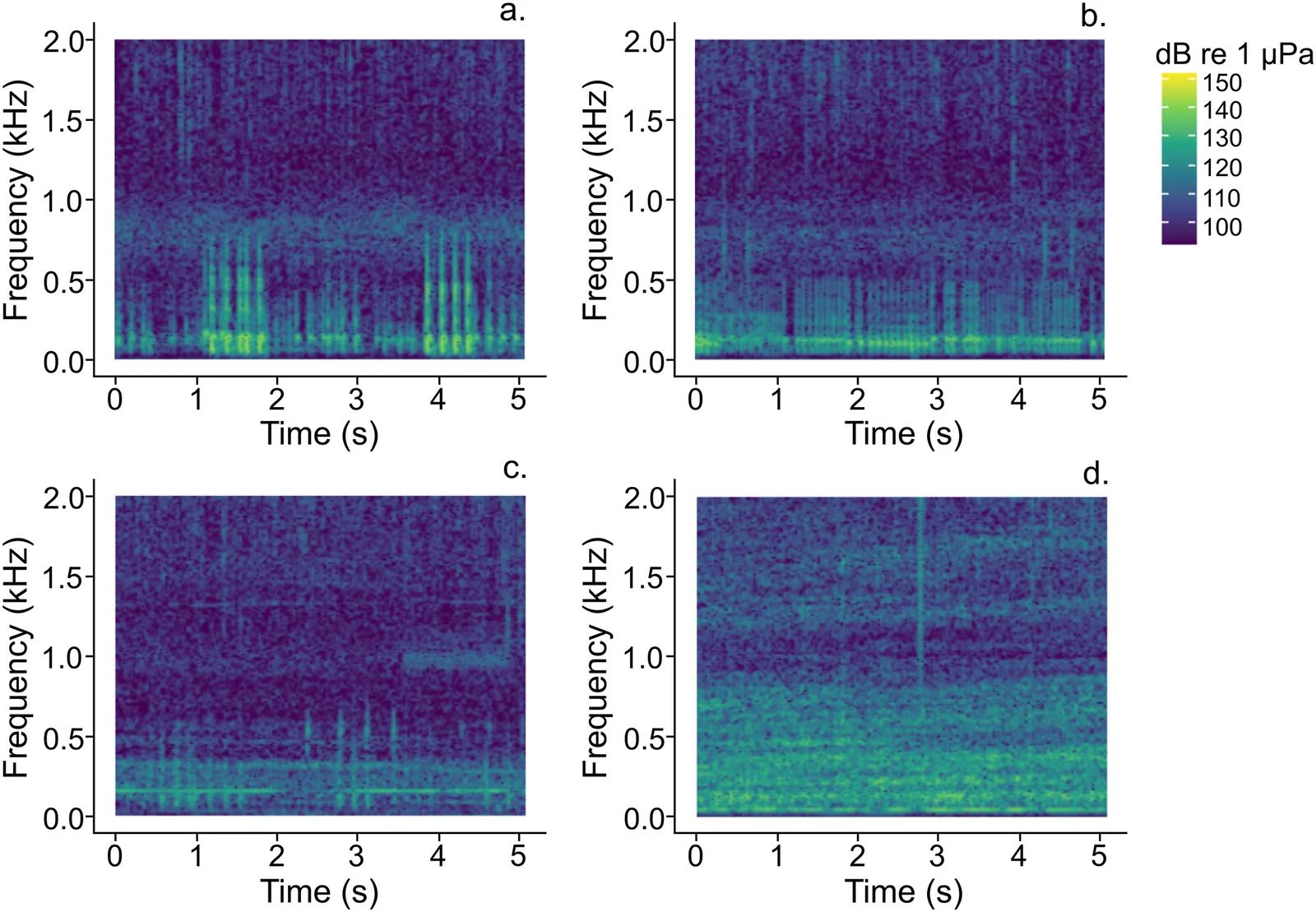
Spectrograms. Spectrograms showing field recordings (0-2kHz) at Saint Andrew Bay, FL. (**a**) Vertical bars showing Red Drum calls in the 0.1-0.9 kHz range. (**b**) Clustered Red Drum calling or “chorusing”. (**c**) Red Drum calls co-occurring with characteristic low-frequency vessel noise (horizontal markings crossing Red Drum calling). High intensity vessel noise (**d**) encompasses a large percentage of the soundscape, which likely obscures Red Drum calls from visual and aural observation.

Vessel noise often obscured the soundscape to a degree that visual (via spectrograms) and aural observations of fish calling were not possible when a vessel was present on the recording (Fig 2). During intense vessel noise, fish may reduce calling because of behavioral disturbance. However, direct assessment of fish calling during intense vessel noise is not possible because this noise would obscure potential calls. If fish maintain the same calling level during vessel noise as in periods where fish calling was recorded but no vessel noise was present, the resulting L_eq_ during vessel noise should be higher than during periods with only fish calling present, as the non-coherent combination of fish sounds and vessel noise would be additive. Observations with vessel noise and potential fish calling (VN/FC) at a lower L_eq_ than prior fish calling by itself would only be expected if fish reduced their calling level during vessel noise, by either lessening calling or moving away from our study site, as predicted by the disturbance hypothesis. However, high amplitude vessel noise (relative to L_eq_ of fish calling alone) could result in vessel noise recordings with higher L_eq_ than periods with fish calling even if fish reduce calling during vessel noise. Thus, to examine the potential of fish modulating their calling level during vessel noise, we focused our analysis on evenings in which fish calling L_eq_ was equal or greater than the median L_eq_ observed during vessel noise. We analyzed 2021 and 2022 separately and used a Wilcoxon sign rank test to determine if L_eq_ during vessel noise periods differed from fish calling L_eq_ measured on the same evenings.

#### Fish calling during and immediately following individual vessel noise events.

We used continuous recordings (Oct. 2022) to examine the acute response of fish calling during and immediately following vessel noise. We predicted that if vessel noise did not alter fish calling, then L_eq_ during periods with vessel noise would exceed L_eq_ measure prior to vessel noise as vessel noise would add acoustic energy to the recording. If calling level during vessel noise lessens in accordance with the disturbance hypothesis, then we would predict that periods with vessel noise could occur at a lower L_eq_ than fish calling L_eq_ prior to vessel noise. Note, however, that if vessel noise exceeds fish chorus calling levels, then periods of combined vessel noise and fish chorus activity will exceed prior fish chorus L_eq_ even if fish calling levels wane.

We identified vessel noise events from continuous recordings and examined L_eq_ of fish calling prior to vessel noise, L_eq_ during vessel noise, and L_eq_ of fish calling immediately after vessel noise. We measured the L_eq_ of fish calling immediately before vessel noise (F1) and immediately after a vessel noise (F2), each from 1 min. of recording data. Vessel noise events were considered as the entire period for which vessel noise was detectable aurally and visually in spectrograms. Each vessel noise event was divided into 20% increments (V1-V5) and L_eq_ was measured for each increment. We restricted observations of vessel noise events to cases with >1 min without vessel noise prior and following each event (F1, F2). We did not include any cases in which the F1 period of calling prior to vessel noise overlapped the F2 of an earlier noise event.

Additionally, a set of 23 cases were used as a control to compare the pre and post fish calling level over the same time frame as the average length of vessel noise (3.5 min.) for these Oct. 022 recordings. These control data consist of 1 min. recordings of Red Drum calling (F1C) followed by a second 1 min. recording beginning 3.5 min. later (F2C).

We used a linear model (LM) to determine if fish calling level changes following vessel noise events. We then used the ‘emtrends’ function of the R package ‘emmeans’ [46] to test the null hypothesis that F1 amplitude would equal F2 amplitude (slope = 1). We used LM models to examine the relationship between F1 amplitude and the amplitude (L_eq_) of the entire vessel noise event (V1-V5), the lowest amplitude among V1-V5 (Vmin), and the highest amplitude among V1-V5 (Vmax). We also used an LM model to examine the relationship between pre and post calling levels from control data (F2C vs F1C) when vessel noise did not occur. We used the ‘emtrends’ function to determine if slopes in each of these additional models differed from a null hypothesis of 1.

## Results

### Temporal overlap of vessel noise and Red Drum choruses

St. Andrew Bay experienced frequent vessel traffic and fish spawning choruses during our study, though their prevalence and the overlap of these two signals varied by month and year. However, recordings during CPs for both spawning seasons captured Red Drum calls (Fig 2a and 2b), vessel noise (Fig 2d), and their co-occurrence (Fig 2c). We observed the highest percentage of CP vessel noise occurrence (45%) in Oct. 2021 (Table 1) while the highest average vessel noise SPL (130 dB) occurred in Oct. 2022. In 2021, 70% of files containing vessel noise also contained some level of Red Drum calls while 58% of vessel noise files had Red Drum calling in 2022. These values likely underrepresent the full extent of masking that occurred in our study site because high intensity vessel noise may have completely masked Red Drum calls on recordings.

**Table 1:** Summary of vessel noise and fish calling by month in 2021 and 2022. Percent of files that contain vessel noise (vessel noise portion, *vnp*); fish calling tendency (*fct*), percent of files without vessel noise in which fish called; noise-free portion (*n-fp*), the percent of files without vessel noise; noise-free fish calling portion (*n-ffcp*), the percent of the total crepuscular period in which fish called and there was no vessel noise; and min. masked, the percent of files in which fish calls were observed co-occurring with vessel noise. Average sound pressure level (SPL dB re 1 µPa) of background noise (BGN), vessel noise (VN), and Red Drum chorusing (RD).

	July 21	Aug.21	Sept.21	Oct.21	July 22	Aug.22	Sept.22	Oct.22
*vnp* (%)	43.7	14.0	30.4	45.1	53.4	37.5	31.3	28.1
*fct* (%)	28.3	54.1	91.4	96.2	34.6	35.3	95.9	100.0
*n-fp* (%)	56.3	86.0	69.6	54.9	46.6	62.5	68.8	69.8
*n-ffcp* (%)	16.5	45.6	63.9	53.6	16.5	23.5	66.0	69.8
*min. masked* (%)	6.1	9.1	27.6	41.8	14.3	13.3	21.9	30.2
BGN (avg. SPL)	116	115	117	116	118	118	121	116
VN (avg. SPL)	117	119	126	125	114	120	125	130
RD (avg. SPL)	114	120	126	126	115	117	126	129
Recording (avg. SPL)	119	120	126	127	122	119	127	130

**Fig 3:**
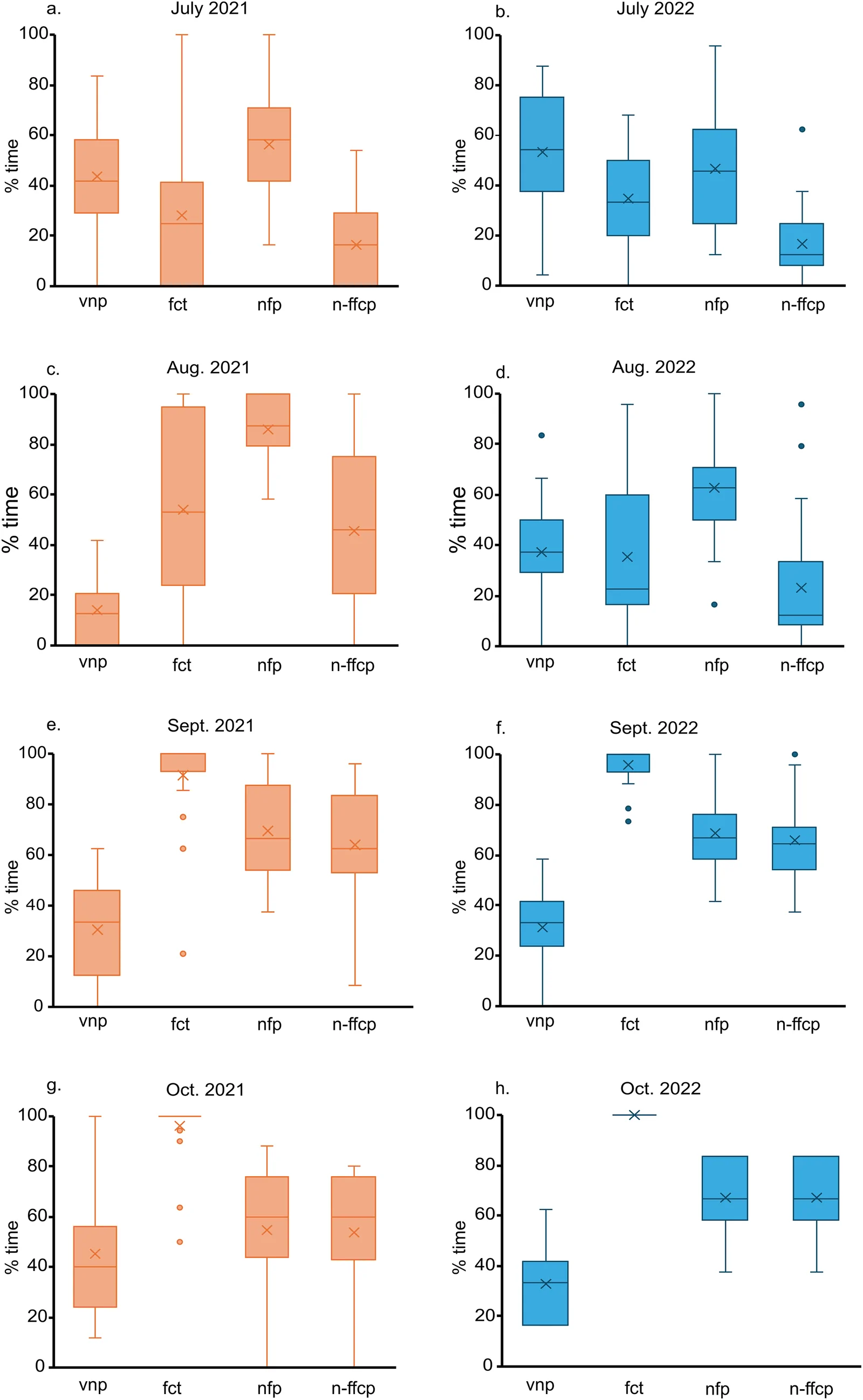
Temporal overlap. Percentage of crepuscular period sound files by month in 2021 and 2022 spawning seasons that contain vessel noise (vessel noise portion, *vnp*), fish calling tendency (*fct*), lack vessel noise (noise free portion, *n-fp*), and contain fish calling (noise-free fish calling portion, *n-ffcp*). Fish calling tendency (*fct*) is the percentage of crepuscular *n-fp* sound files containing fish calls. Boxes indicate 25^th^-75^th^ percentile, line indicates the median, x indicates the mean, whiskers encompass data range excluding outliers, dots are outliers.

Monthly vessel noise occurrence (vnp) in the CP ranged from 14% in August (Fig 3c.) to 45.1% in October (Fig 3g) in 2021 and 31.3% in September (Fig 3f) to 53.4% in July (Fig 3b) in 2022. Two days in 2021, October 9–10, contained vessel noise in 100% of the recordings during the CP. The highest percentage of the CP to display vessel noise in 2022 was 87.5% on

July 23. In both spawning seasons, Red Drum fish calling tendency (fct), the percentage of the noise free portion (n-fp) (non-vessel noise portion of the CP) to contain fish calling, was higher in late-summer and early autumn at over 90% in September-October (Fig 3e–3h). Note that this period of high fct largely corresponds with the period for which high fish calling L_eq_ were observed, days 75–100 (13 September – 8 October) in both years.

### Effects of vessel noise and other abiotic variables on fish calling

**Fig 4:**
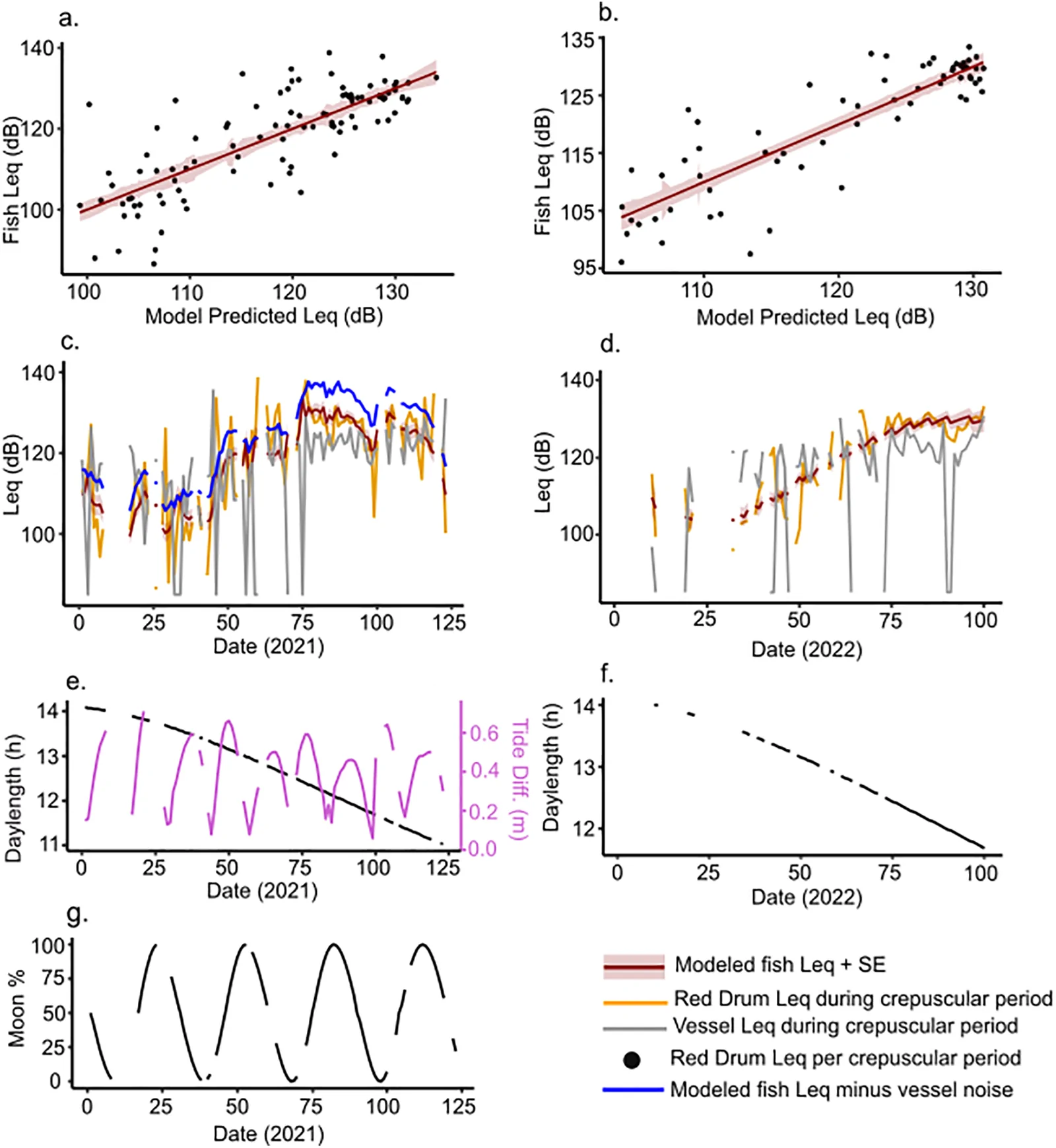
Nightly calling. Red Drum call sound level equivalent (L_eq_) (dB re: 1µPa^2^·s) over a daily two-hour crepuscular period with Generalized Least Squares models used to determine its association with vessel noise and other abiotic variables. (**a**) Best-fit model for 2021 spawning season included vessel L_eq_, moon phase, tidal differential, and an interaction of date and daylength. (**b**) Best-fit model for 2022 included an interaction of date and daylength but did not include vessel L_eq_. (**c**) Fish call L_eq_ (gold) and vessel (grey) over the 2021 spawning season along with the fitted line (red) and the model without vessel as a predictor (blue). (**d**) Fish call L_eq_ (gold) and vessel (grey) over the 2022 spawning season along with the fitted line (red). (**e**) Daylength (black) and tidal differential (purple) over 2021 spawning season. (**f**) Daylength over 2022 spawning season. Note for 2021, the model without vessel noise included would predict elevated levels for fish L_eq_ (blue line). (**g**) Moon phase (illumination) over 2021 spawning season.

Evening Red Drum sound production was influenced by multiple abiotic factors and with vessel noise. In 2021, Red Drum total crepuscular calling L_eq_ by date was negatively correlated with evening vessel L_eq_, after inclusion of abiotic variables that were most strongly predictive (Table 2 and Fig 4). The interaction of date and day length was the most supported predictor in the model (Table 2). This interaction predicts an increase in calling associated with the reduction of daylength, but with calling peaking (highest evening L_eq_ value, September 15, 2021, October 4, 2022) prior to the shortest day of the year of our study period. Additionally, calling was positively correlated with moon illumination and tidal differential during the 2021 spawning season. In 2022 there was also an interactive effect between date and daylength with these acting as the sole predictor of total crepuscular calling L_eq_ by date (Table 2 and Fig 4). Notably, 2022 recording period was shorter than 2021 with the last half of the month of October not being collected. Additionally, the auto regressive model was supported in 2022 but not for 2021. This indicates Red Drum calling L_eq_ was more clustered over successive days in 2022.

**Table 2:** GLS Models for crepuscular periods over two spawning seasons (2021-2022). Selected model appears in bold. AR = autoregression, Non-AR = non-autoregression, Tide diff. = tidal differential. Bold text emphasizes the most parsimonious model for each year.

Year	AR Model	Model AICc	Δ AICc	Terms	ΔAICc to remove	Coeff.	SE	p-value	d.f.
**2021**	**Non-AR**	**706.1**		**Intercept**		**2075.49**	**286.74**	**<0.001**	**100, 93**
				**Date**		**−7.69**	**1.08**	**<0.001**	
				**Daylength**		**−139.17**	**20.17**	**<0.001**	
				**Moon phase**	**6.58**	**0.07**	**0.02**	**0.004**	
				**Tide diff.**	**3.83**	**11.87**	**4.87**	**0.016**	
				**Vessel**	**2.94**	**−0.05**	**0.02**	**0.026**	
				Date*Daylength	**42.85**	**0.38**	**0.05**	**<0.001**	
	AR(1)	706.3	0.14	Date + Daylength + Vessel + Moon phase + Tide diff. + Date*Daylength					
	AR(2)	708.3	2.12	Date + Daylength + Vessel + Moon phase + Tide diff. + Date*Daylength					
	Non-AR	789.4	83.21	Null					
	AR(1)	791.1	84.98	Null					
	AR(2)	793.1	86.93	Null					
**2022**	**AR(1)**	**406.4**		**Intercept**		**2320.35**	**679.05**	**0.001**	**65, 61**
				**Date**		**−8.39**	**47.66**	**0.002**	
				**Daylength**		**−155.94**	**2.79**	**0.004**	
				Date*Daylength	**2.79**	**0.40**	**0.14**	**0.005**	
	Non-AR	479.6	73.2	Temp.					
	AR(2)	406.9	0.46	Date					
	Non-AR	497.2	90.78	Null					
	AR(1)	418.6	12.19	Null					
	AR(2)	411.6	5.16	Null					

### Prior exposure impact on Red Drum calling

**Fig 5:**
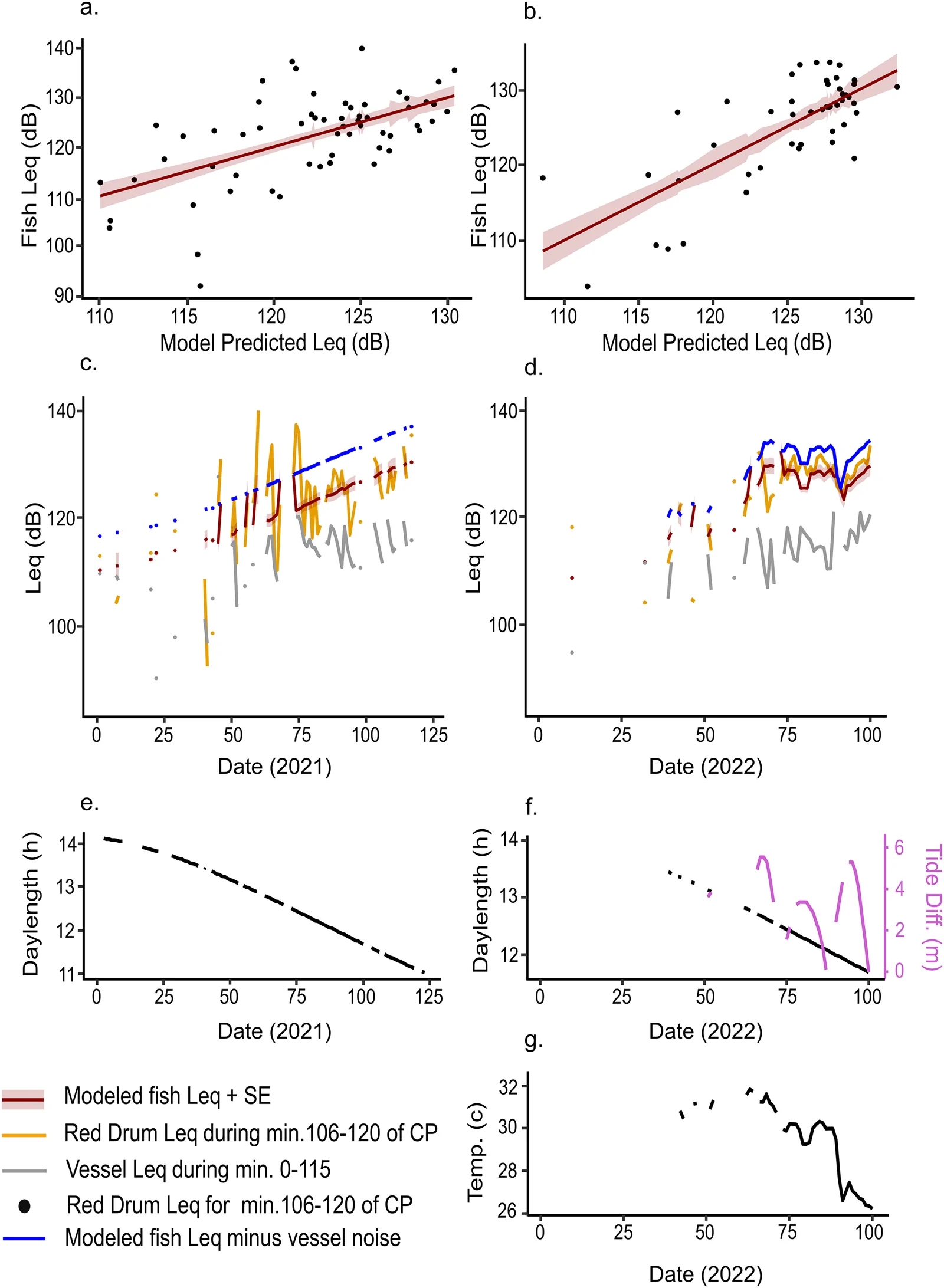
Prior noise impact. Prior vessel noise predicts quieter Red Drum equivalent sound level (dB re: 1µPa). Best fitted models for Red Drum calling level (Leq) in the final 15 min portion of the crepuscular period predicted by vessel noise and daily abiotic variables in the prior 105 min for 2021 (**a**) and 2022 (**b**). For 2021 prior vessel L_eq_ and daylength were both included in the best fit model. For 2022 prior vessel L_eq_, daylength, moon phase, and temperature were all included in the best fit model. (**c-d**) Vessel L_eq_ (grey) in the initial 105 min of the crepuscular period (CP), Red Drum call L_eq_ (gold) for the final 15 min of the CP and the fitted line (red) over both 2021 and 2022 spawning seasons along with the model minus vessel noise (blue). (**e-g**) Abiotic predictors for both spawning seasons; daylength (**e**) 2021, daylength, moon phase (purple), and temp. (**f, g**) 2022. Note that for both 2021 and 2022, the model without vessel noise included predicts elevated fish L_eq_ (blue line).

In accordance with expectations of the disturbance hypothesis, prior exposure to vessel noise exposure during the first 105 min. of the CP predicted a reduced fish calling level during the final 15 min. of the CP for both 2021 and 2022 (Fig 5 and Table 3). This indicates that the level of fish calling observed in the final 15 mins of the CP was negatively correlated with the L_eq_ of vessel noise during the 105 min. prior. Additionally, day length correlated negatively with Red Drum L_eq_ during both spawning seasons. Moon phase and temperature were both positively correlated with fish L_eq_ in 2022 only (Table 3 and Fig 5f and 5g).

**Table 3:** GLS models for final 15 min of crepuscular period over two spawning seasons (2021-2022). Selected model appears in bold. AR = autoregression, Non-AR = non-autoregression, Tide diff. = tidal differential. Bold text emphasizes the most parsimonious model for each year.

Year	AR Model	Model AICc	Δ AICc	Terms	ΔAICc to remove	Coeff.	SE	p-value	d.f.
**2021**	**AR(2)**	**401.2**		**Intercept**		**216.27**	**18.42**	**<0.001**	**59, 56**
				**Boat Prior**	**2.98**	**−0.05**	**0.02**	**0.022**	
				**Daylength**	**11.85**	**−7.06**	**1.42**	**<0.001**	
	Non-AR	410.4	9.27	Boat Prior + Daylength					
	AR(1)	404.7	3.51	Boat Prior + Daylength					
	Non-AR	433.3	19.78	Null					
	AR(1)	413.5	12.31	Null					
	AR(2)	414.0	3.55	Null					
**2022**	**Non-AR**	**294.2**		**Intercept**		**238.48**	**16.68**	**<0.001**	**48, 43**
				**Boat Prior**	**2.50**	**−0.04**	**0.02**	**0.033**	
				**Daylength**	**6.70**	**−16.42**	**2.35**	**<0.001**	
				**Temp.**	**14.48**	**0.07**	**0.02**	**0.004**	
				**Moon phase**	**33.44**	**3.09**	**0.72**	**<0.001**	
	AR(1)	296.1	1.97	Boat Prior + Daylength + Moon + Temp.					
	AR(2)	298.1	3.95	Boat Prior + Daylength + Moon + Temp.					
	Non-AR	324.9	30.78	Null					
	AR(1)	310.0	15.81	Null					
	AR(2)	307.9	13.78	Null					

### Fish calling during vessel noise compared to periods without vessel noise

**Fig 6:**
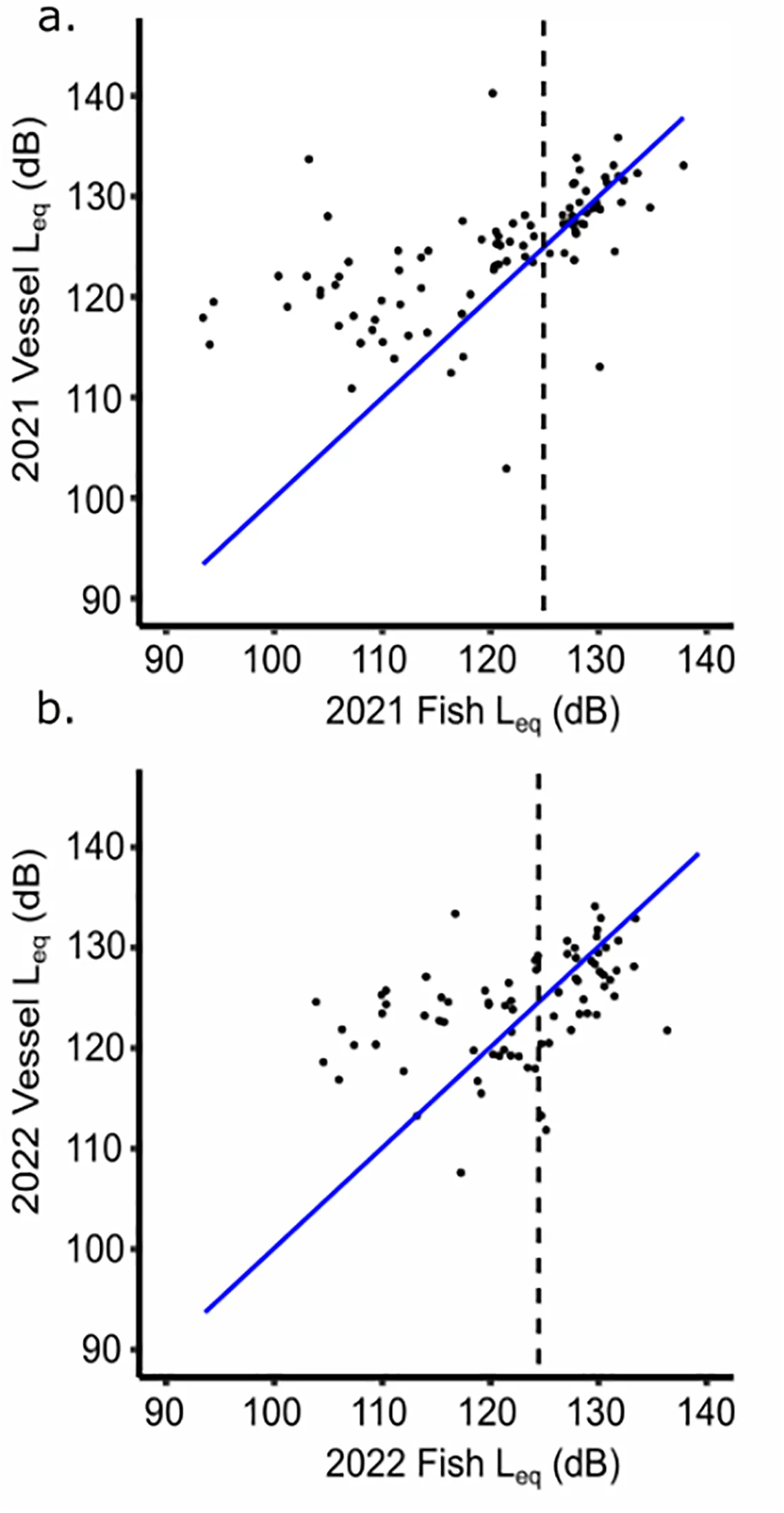
Possible fish L_eq_. Fish calling L_eq_ vs. vessel noise L_eq_ by night in 2021 (**a**) and 2022 (**b**). Points below the blue line are consistent with predictions of the disturbance hypothesis as these show vessel noise levels with a lower L_eq_ than fish calling during the same CP without vessel noise. This would only be possible if the fish calling level during vessel noise was lower compared to the calling level when vessel noise was not present because the combined noncoherent signals would be additive. Points above the 1:1 line are ambiguous because it is impossible to assess the contribution of fish calling because it is possible that vessel noise occurred at an amplitude higher than fish calling by itself. Blue line represents a 1:1 relationship. Dash line is median vessel noise amplitude.

Impacts from vessel noise were obscured on evenings with low amplitude fish calling; however, on evenings when calling exceeded median vessel noise levels, fish sound level was shown to decrease or remain consistent with non-vessel periods. In both 2021 and 2022, when fish calling L_eq_ was low to moderate, L_eq_ during vessel noise periods regularly exceeded fish calling L_eq_ (Fig 6). In these instances, it is not possible to determine if fish calling L_eq_ changed during vessel noise because there is a greater likelihood of vessel noise amplitude exceeding the amplitude of fish calling alone. In 2021, on evenings when fish calling L_eq_ was > 125.1 dB (median 2021 vessel noise amplitude), L_eq_ during vessel noise periods did not differ (n = 36, p = 0.499, Wilcoxon sign rank test) from fish calling L_eq_ (Fig 6A). In 2022, when fish calling L_eq_ was > 124.6 dB (median 2022 vessel noise amplitude), L_eq_ during vessel noise periods was lower than fish calling L_eq_ (2.5 dB median difference, 0.5–4.9 dB quartile 1-quartile 3) measured on the same evening (n = 35, p < 0.001, Wilcoxon sign rank test) (Fig 6B). Lower L_eq_ values during vessel noise periods is consistent with the expectations of the disturbance hypothesis.

### Fish calling during and immediately following individual vessel noise events

**Fig 7:**
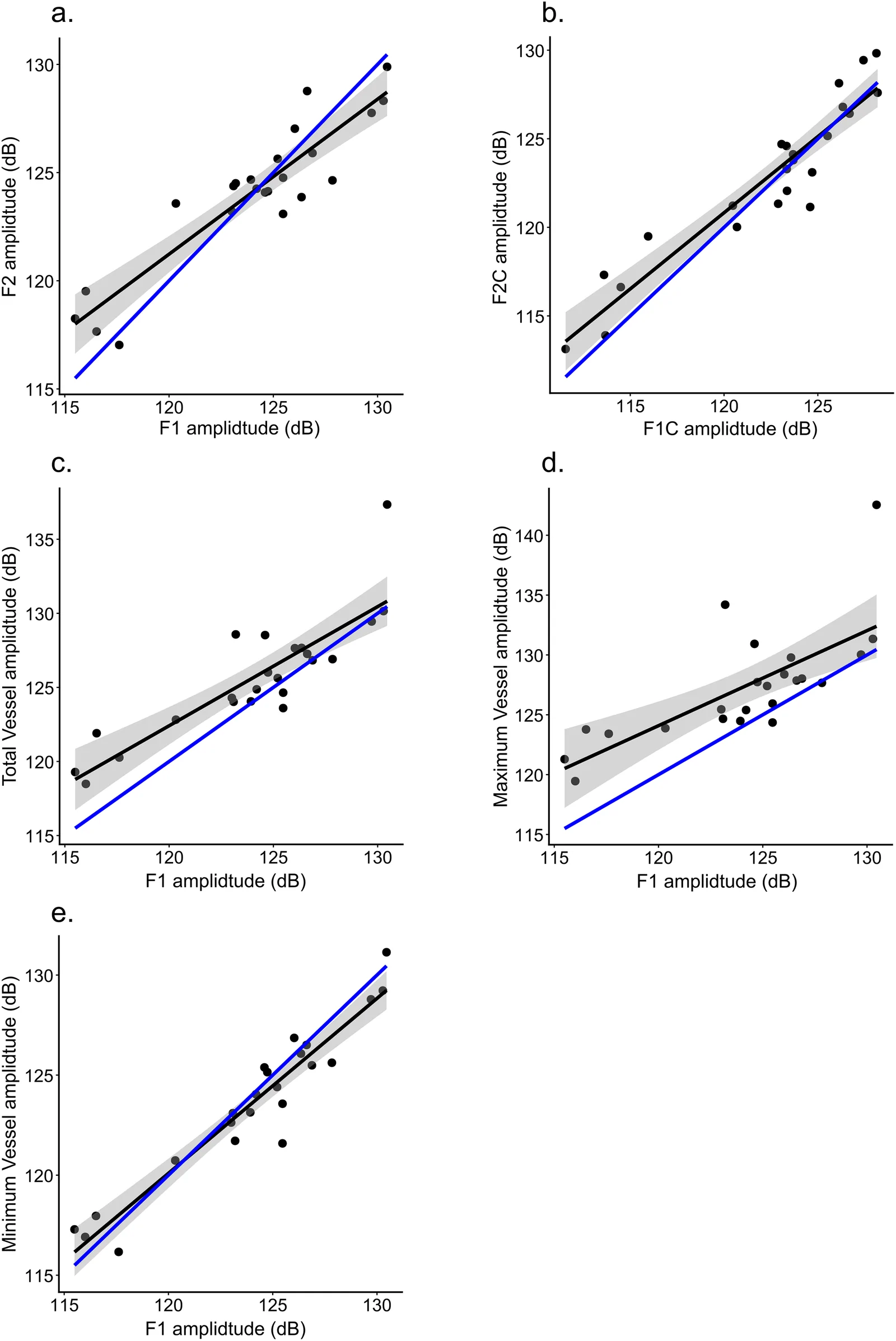
Immediate impact. Linear Models (black line, gray band = 95% CI) showing the relationship between (**a**) fish calling level prior to vessel noise (F1) and fish calling level after vessel noise (F2), (**b**) prior (F1C) and following (F2C) fish calling levels under control conditions without intervening vessel noise, (**c**) fish calling level prior to vessel noise (F1) and sound level during the entire period (V1-V5) of intervening vessel noise, (**d**) fish calling level prior to vessel noise (F1) and the value of the highest amplitude segment of intervening vessel noise (V1-V5), and (**e**) fish calling level prior to vessel noise (F1) and the value of the lowest amplitude segment of intervening vessel noise (V1-V5). All models are presented alongside a 1:1 line (blue) representing a hypothetical amplitude unchanged from the initial fish calling level.

Red Drum calling levels differed following vessel noise events (Fig 7A). A LM model examining the relationship between F1 and F2 amplitudes indicated a slope less than (0.72, p < 0.001). The LM model indicated that when fish call at low levels prior to vessel noise, they were more likely to increase their calling level following vessel noise (in accordance with the compensation hypothesis), but at high initial calling levelss fish were more likely to call at a lower level following vessel noise (an expectation of the disturbance hypothesis). In contrast, the relationship between fish calling before and after periods without vessel noise (control; Fig 7B) had a slope that did not differ significantly from 1 (0.82, p = 0.050). In this control comparison, lower initial calling levels tended to be followed by higher subsequent calling levels (Fig 7B). Higher initial calling levels, however, tended to be followed by similar subsequent calling levels (Fig 7B).

Total vessel noise level (L_eq_ of V1-V5) tended to exceed prior fish calling level (F1) (Fig 7c). The slope between F1 and total vessel noise did not differ significantly from 1 (0.80, p = 0.074). Similarly, the maximum amplitude of vessel noise segments V1-V5, exceeded prior fish calling levels F1 (Fig 7d), with a slope that did not differ significantly from one (0.79, p = 0.229). During the lowest amplitude portion of vessel noise V1-V5, however, amplitudes tended to be lower than the prior fish calling level (F1), particularly when F1 calling levels were greatest (Fig 7e). Notably, the slope of the relationship between F1 and minimum vessel noise portion amplitude was significantly less than one (0.87, p = 0.049) because of the relatively low residual variation around the fitted model compared to models when vessel noise was greater. This observation indicates that at least during these time periods with weaker vessel noise, fish chorus levels wane compared to prior F1 levels.

## Discussion

The findings of our study indicate potential disturbance of Red Drum spawning aggregation calls during vessel noise. The disturbance hypothesis was supported, with data reflecting lower total crepuscular fish call L_eq_ in association with total crepuscular vessel noise in 2021. Evidence of disturbance from total crepuscular fish call L_eq_ in association with vessel noise was not observed in 2022, but the recording period was shorter for 2022 and did not capture behavior of Red Drum as late into the season as 2021.

L_eq_ of vessel noise 105 min prior to the end of the CP was shown to have a negative correlation with L_eq_ of fish in the final 15 min. of the CP. These results, in support of disturbance hypothesis, were observed in both 2021 and 2022, when lower fish call L_eq_ was associated with higher L_eq_ of anthropogenic noise in the preceding period. This analysis shows that the impact of vessel noise is not just an immediate issue, as prior vessel noise exposure can also affect fish behavior. Lower call levels observed in this analysis at the end of the CP indicate that Red Drum do not acclimate to noise exposure over the CP. This acute response in call level could be due to reductions in the number of calls or duration of calling by individual fish, or fish may vacate habitat with increased vessel noise. This contrasts with research in the Gulf of Mexico off Florida which found no evidence of vocal modification in response to ferry noise in a closely related species, Atlantic Croaker (*Micropogonias undulatus*) [47].

Notably, without visual confirmation of fish proximity, we are unable to determine if fish remain near the recorder. This means our findings are always framed as the measurable amount of fish sounds within the recording area compared to vessel sounds in the same vicinity. We are unable to confirm how fish respond beyond reporting total acoustic signal above background noise. However, we are able to estimate the total signal amplitude received by our recorder and establish an estimated SNR for fish and vessels in the same approximate location and compare the two.

The use of a single hydrophone, and not an array, also prevented us from determining the location of fish calling or vessels in the recording area. Because of this we cannot state if fish vacate the recording area or lessen/terminate calling when we observed a decrease in fish calling in response to vessel noise. In spite of this, we do not believe our findings are due to fish vacating habitat alone for multiple reasons. Firstly, Red Drum do not appear to permanently vacate the area following noise exposure as Red Drum sounds were recorded over the CP throughout the spawning season, over consecutive nights, even though vessel noise was ubiquitous with fish calling cooccurring in 70% of recordings with vessel noise in 2021 and 58% in 2022.

Interestingly, the sciaenid Brown Meagre (*Sciaena umbra*) was found to occur in noisy habitats near Venice Lagoon, and less so in quieter habitats which indicates that factors other than vessel noise likely exert a stronger influence on habitat choice [48]. Additionally, fish may have other means of signaling beside acoustic communication that they employ when background noise becomes too intense. Two gobiids were observed in lab settings to reduce spawning sounds but continued with visual communication in the presence of increased background noise [8]. These gobiid fishes still spawned in the presence of anthropogenic noise, though were forced to deviate from their typical behaviors. Perhaps behavioral plasticity exists in other species, though in turbid environments, such as those often inhabited by nearshore fishes of the Gulf of Mexico, visual communication would likely be less effective.

During our study, on evenings with the highest fish calling L_eq_, we observed evidence that fish calling L_eq_ decreased, which could result from fewer total fish calling, a reduction in call rate, or fish moving away from the recording area during vessel noise, consistent with predictions of the disturbance hypothesis. In 2022, when fish calling L_eq_ was as high or greater than the median L_eq_ of vessel noise periods, L_eq_ during vessel noise tended to be lower than fish calling L_eq_ from the same evening. If fish were to maintain or increase their calling level, recordings with vessel noise over the same evening should exceed fish calling L_eq_ alone as the two signals should be additive, resulting in a higher amplitude than a single signal [49]. This predicted disturbance response can only be tested when fish calling L_eq_ is greater or equal to vessel L_eq_ because an effect cannot be detected when fish call at amplitudes lower than the concurrent vessel noise. However, we are unable to determine if fish calling L_eq_ is modified when L_eq_ during vessel noise presence exceeds L_eq_ of fish calling alone because this could result either when vessel noise amplitude exceeds fish calling L_eq_ or when fish calling and vessel noise combined result in a higher amplitude than fish calling alone. In 2021, when fish calling L_eq_ was greater or equal to the median L_eq_ of vessel noise periods, L_eq_ of fish calling were not found to differ from L_eq_ of vessel noise periods; i.e., there was neither evidence of a reduction (disturbance) in fish calling during vessel noise nor evidence of an increase in fish calling during vessel noise (compensation). Notably, L_eq_ during vessel noise was slightly higher in 2021 compared to 2022 (median 125.1 vs. 124.6 dB) and fish calling L_eq_ in 2021 was slightly lower compared to 2022 (median 121.5 vs. 124.1 dB). Thus, there may be a greater opportunity to test predictions of the disturbance hypothesis when fish calling levels are higher than received vessel noise amplitudes, which may have been the case in 2022. Also, it should be noted Red Drum may respond differently between high or low intensity vessel noise or when calling at lower sound levels, i.e., compensate under low levels of vessel noise and act disturbed under higher vessel noise.

Reductions in calling during vessel noise predicted by a disturbance hypothesis are reported for several sciaenid fishes: Meagre (*Argrosomus regius*) calling during vessel noise in the Tagus estuary of Portugal [9], and *Pogonias courbina*, decreased call rate during vessel noise in Argentina [50]. These findings also align with preliminary analysis of a subset of our data set showing disruption of Red Drum calling by vessel noise [51]. The results of our study, which expands the scope of preliminary work, and other recent investigations indicate that sciaenid behavioral responses to vessel noise may vary by location and among species due to population differences, environmental factors, or a combination of both [52]. In the present study, data from continuous recordings indicated that when calling levels prior to vessel noise were high, subsequent calling levels immediately after vessel noise was lower. When prior calling levels were low, subsequent calling levels after vessel noise tended to be higher. The slope between prior and subsequent calling was less than one and consistent with the prediction of the disturbance hypothesis that, at least at high calling levels, fish may reduce calling following vessel noise. It should be noted, however, that in control data, the observed slope between prior and subsequent calling was also less than one, though not significant. The less than one slope relationship of prior and subsequent calling may occur because at low initial calling levels, higher subsequent calling levels are more likely because lower levels would drop below the noise floor and at high initial calling levels, higher subsequent calling levels are not possible because of constraints on fish being able to call at a higher level and call rate. Based on our analysis of sound amplitude during the lowest amplitude portion of intervening vessel noise, it appears that when the initial fish calling level is high, the fish calling level is reduced during vessel noise. Further investigation is warranted, particularly in environments where vessel noise is not as intense and so may not obscure the contribution of fish calling to the potentially combined vessel and fish L_eq_. It is conceivable that fish may respond differently based on the intensity of background noise, e.g., maintain or even increase calling amplitude when vessel noise is low (no response or compensation) and reduce calling when vessel noise is more intense (disturbance). For future studies, an experimental approach with a known vessel noise source that passes repeatedly over a study area with the same path and speed that could be measured in the absence of fish calling (e.g., earlier in the day before chorusing occurs) and during choruses, could test these responses but would also require that other vessel noises are not present, which would be a challenge at this study site.

Our analyses assumed that Red Drum were randomly dispersed around the hydrophone and thus over the course of each study night, the chance of detection and loss from attenuation with distance was not correlated with vessel noise. We routinely observed adult Red Drum near the recorder during dives to deploy and service our equipment. We were not able to directly estimate signal attenuation and detection range in our study, which would provide an estimate of received level variation associated with fish movement away from the hydrophone when call rate is constant. Because the study site is in relatively shallow water, attenuation from geometric spreading (ignoring absorption) is expected to fall between cylindrical (SL = RL + 10·log_10_R) and spherical spreading (SL = RL + 20·log_10_R), where SL = source level, RL = received level, and R = source distance from the hydrophone. Using our minimum detectable received level of Red Drum choruses (108 dB) and a potential high amplitude source level of 150 dB [25], there is wide variation in the detection radius predicted by spherical spreading (126 m) and cylindrical spreading models (15.8 km).

During most of the spawning season, received L_eq_ varied more modestly (10–20 dB). Reductions in received L_eq_ from calling fish in the present study following periods of vessel noise could result from fish moving further away from the recorder. A 10 dB reduction in received level would result if fish were present at the source and subsequently moved away (3.2 m under spherical spreading or 10 m under cylindrical spreading). Fish initially detected at a moderate distance from the hydrophone, however, would need to move much further away from the source to result in a 10 dB reduction. Future studies with a synchronized hydrophone array could be used to reduce error associated with source (fish and vessel sound) position for Red Drum and potentially determine if calling fish move away from noise sources or simply alter call rate [33].

Analyses used in our study assumed that sound amplitudes combined from multiple sources (individual fish, vessels, and background noise) did not combine constructively or destructively. This was based on the observation that Red Drum calls are trains of short duration pulses separated by a silent interval, rather than tonal signals. Thus, we assumed these sources combined additively as predicted for non-coherent signals [49], as has been assumed in other field studies that examined fish sound production [53,54]. Use of a hydrophone array in future studies may shed light on how fish and vessel sounds combine at different locations within the estuary to determine the validity of these assumptions.

In our study, we did not determine vessel noise source level and location. Source levels and frequencies vary widely depending on vessel type and operation [11]. Our aim was not to determine noise level from individual vessels, but rather to determine the received sound level at the recorder location from all vessel noise sources regardless of the number of vessels, operation, and source location. Our recordings represent what Red Drum may be exposed to in the study site by observing noise pollution present regardless of source. Future work with non-duty cycled recordings and hydrophone arrays is needed to better assess how source levels from individual vessels influence spatial patterns of Red Drum calling. This would allow for a more comprehensive understanding of the spatial patterns of vessel noise and fish calling and could potentially determine if calling fish move in response to vessel noise. Another consideration for future studies would be the measurement and characterization of particle motion levels to determine the impact of particle motion on fish responses to passing vessels. Particularly in the near field, particle motion is believed to be a major component of received auditory information in fishes [55–57]. While our study focused on the contribution of sound pressure from vessel noise and how it impacted choruses of Red Drum aggregations, further research could investigate how particle motion from noise pollution plays a role in impacting spawning behavior.

Though we observed reduced Red Drum call L_eq_ during and in association with vessel noise, this pollution source is a relatively recent potential selection pressure for the evolution of sound production behavior in fishes and evidence exists that some sciaenids, along with other taxa, do not avoid noisy waterways or alter calling behavior during vessel noise [13,48]. Brown Meagre showed an increase in call rate after repeated vessel noise exposure [6]. Plainfin Midshipman (*Porichthys notatus*) and Oyster Toadfish (*Opsanus tau*), both batrachoidids, increase their call amplitude when artificial noise is introduced to calling males [58,59]. In addition to increasing amplitude, Plainfin midshipman also lower their call frequency, potentially to avoid masking by noise pollution [59]. Freshwater Drum (*Aplodinotus grunniens*) may also vary their peak call frequency in the presence of vessel noise when calling at high amplitudes [60]. Though a frequency shift was not measured in our study, if Red Drum do shift call frequency, it would be highly unlikely that such a shift would occur outside the 0–600 Hz band observed. Additionally, there exists the potential that fish may have shifted sound production to another time outside the CP. Though in situ observations have previously shown Red Drum calling to peak after sunset [20,26], it is conceivable that fish may use other times to call when vessel noise is not as intense. When recording aquacultured Red Drum, researchers noted fish calling peaked in the morning hours [61], though this was not attributed to disturbance from noise pollution. Though this is outside the context of our study, assessing the full 24h cycle of sound production in the face of vessel noise may be worth researching in the future.

This research also adds to the ever-growing literature that shows anthropogenic noise is highly prevalent in marine coastal habitats [3,10,62]. We documented significant periods of noise pollution in the environment with fish sounds over repeated spawning seasons (Fig 7). Noise pollution has been seen in other aquatic systems and is concerning in its prevalence. In May River, South Carolina researchers saw a 21% overlap in time for Red Drum calls and vessel noise [31]. Notably in Oct. 2021, our study showed on average 100% of the CP contained either vessel noise or Red Drum calling, which means every opportunity for fish to call without potential masking of their calls was used. This leads to the reasonable conclusion that significant overlap in time exists between vessel noise and when Red Drum calling occurs in Saint Andrew Bay. Beyond the potential for altered acoustic behavior, this excessive noise exposure could present further risk to Red Drum and other marine life.

## Conclusion

Our study showed Red Drum spawning choruses lessened in association with elevated vessel noise in Saint Andrew Bay, FL and that significant overlap of vessel noise and Red Drum calling exists in this habitat. We also show that noise exposure is not only acute in its impact, but that prior cumulative exposure has a relationship with decreased calling later in the day. In light of these findings, continued investigation of vessel noise impacts on sciaenid calling behavior is needed, with both lab experimentation and in situ studies. Assessment of sound production by individual fish or small groups in controlled environments will allow for greater insights into how these animals respond to noise pollution. Furthermore, though our research focused on the impact of low frequency noise pollution (0–600 Hz), future studies should assess how the contribution of high frequency, broadband noise in the environment, produced by many vessels, may impact animal behavior. Ongoing use of passive acoustic monitoring in coastal regions is needed to elucidate multispecies responses to the ever-growing noise pollution found in these regions. A comprehensive understanding of noise pollution impacts on aquatic life is critical for coastal ecosystem management.
